# Sensitization by subcutaneous route is superior to intraperitoneal route in induction of asthma by house dust mite in a murine mode

**DOI:** 10.1590/S1679-45082015AO3389

**Published:** 2015

**Authors:** Marcelo Vivolo Aun, Beatriz Mangueira Saraiva-Romanholo, Francine Maria de Almeida, Thayse Regina Brüggemann, Jorge Kalil, Milton de Arruda Martins, Fernanda Magalhães Arantes-Costa, Pedro Giavina-Bianchi

**Affiliations:** 1 Faculdade de Medicina, Universidade de São Paulo, São Paulo SP, Brazil.

**Keywords:** Models, animal, Asthma/chemically induced, Pyroglyphidae

## Abstract

**Objective:**

To develop a new experimental model of chronic allergic pulmonary disease induced by house dust mite, with marked production of specific immunoglobulin E (IgE), eosinophilic inflammatory infiltrate in the airways and remodeling, comparing two different routes of sensitization.

**Methods:**

The protocol lasted 30 days. BALB/c mice were divided into six groups and were sensitized subcutaneously or intraperitoneally with saline (negative control), *Dermatophagoides pteronyssinus* (Der p) 50 or 500mcg in three injections. Subsequently they underwent intranasal challenge with Der p or saline for 7 days and were sacrificed 24 hours after the last challenge. We evaluated the titration of specific IgE anti-Der p, eosinophilic density in peribronchovascular space and airway remodeling.

**Results:**

Both animals sensitized intraperitoneally and subcutaneously produced specific IgE anti-Der p. Peribronchovascular eosinophilia increased only in mice receiving lower doses of Der p. However, only the group sensitized with Der p 50mcg through subcutaneously route showed significant airway remodeling.

**Conclusion:**

In this murine model of asthma, both pathways of sensitization led to the production of specific IgE and eosinophilia in the airways. However, only the subcutaneously route was able to induce remodeling. Furthermore, lower doses of Der p used in sensitization were better than higher ones, suggesting immune tolerance. Further studies are required to evaluate the efficacy of this model in the development of bronchial hyperresponsiveness, but it can already be replicated in experiments to create new therapeutic drugs or immunotherapeutic strategies.

## INTRODUCTION

Asthma is an inflammatory disease of the airways associated with bronchial hyperresponsiveness, which leads to recurring episodes of cough, dyspnea, wheezing, and chest tightness.^(1,2)^ The pathogenesis of allergic asthma, which accounts for up to 70% of cases, involves exposure to allergens, the phenomenon of sensitization, and the consequent type I hypersensitivity reaction, mediated by immunoglobulin E (IgE).^(3,4)^ However, the immunopathological mechanism, initially mediated by IgE, is a lot more complex, including diverse cells, inflammatory mediators, and cytokines, with a predominance of helper T type 2 lymphocytes (TH2 cells).^(5)^ The complexity of the pathogenesis of human asthma hinders establishing experimental models with animals that faithfully reproduce the clinical forms found in daily practice.^(1,6,7)^

An understanding of the pathological mechanisms, of the critical pathways involved, and the targets for new therapeutic strategies would be ideally determined by studies in human beings.^(6,7)^ However, due to obvious ethical issues, the studies necessary for such conclusions are not performed in asthmatic subjects.^(6)^ Hence, the formulation of simple and effective animal models that do not demand high costs is indispensable.

The most commonly used animals in asthma models have been rodents, especially mice and rats, due to their greater availability, cost-effectiveness, and easy manageability.^(6)^ Most asthma models with mice use ovalbumin as foreign sensitizing protein associated with an adjuvant, especially aluminum hydroxide.^(7)^ Nevertheless, more recent models have used ovalbumin without adjuvant for sensitization, with results comparable to those of the classic models, with alum.^(8)^

The most frequently used route for sensitization is intraperitoneal (IP), generally with two injections, separated by a period of 7 to 14 days, and normally there is a new exposure to the antigenic protein directly in the airway, by inhalator route (aerosol), or intranasal (IN) or intratracheal installation.^(7)^ However, the clinical significance of models with ovalbumin to mimic human asthma has been questioned. Therefore, models with house dust mites, pollen, cockroaches, or fungi, which are known aeroallergens involved in the pathogenesis of human allergic asthma, have been used.^(7,9)^ Aeroallergens have already been studied for sensitization and challenge by different routes and with varied doses.^(10-13)^ Our group also demonstrated that different species of mice can present with antagonist responses in dose-response curves of sensitization to the house dust mite *Dermatophagoides pteronyssinus* (Der p) in a model of allergic conjunctivitis.^(14)^ Nevertheless, one of the great difficulties in setting up effective models of asthma has been the chronicity of the process, with development of the disease mediated by IgE, with pulmonary eosinophilia, but that also causes remodeling of the airways.

## OBJECTIVE

To develop a new experimental model of the chronic allergic pulmonary disease induced by house dust mite, with prominent production of specific IgE, inflammatory eosinophilic infiltrate in the airways, and remodeling, and to compare the two different sensitization routes and two distinct doses of dust mites in this process.

## METHODS

Specific-pathogen-free (SPF) male adult BALB/c strain mice were used, aged six to eight weeks. The animals came from the Central Animal Laboratory of the *Faculdade de Medicina da Universidade de São Paulo* [Medical School]. The mice were kept in the maintenance animal laboratory of the *Laboratórios de Investigação Médica* (LIM) [Medical Investigation Laboratories] 05 and 20, with food and water *ad libitum*. All animals received care as per the Guide for the Care and Use of Laboratory Animals*,* published at the National Institutes of Health (NIH; publication 85-23, revised in 1985), and all experiments were performed under general anesthesia. The protocol was approved by the Research Ethics Committee of the *Faculdade de Medicina da Universidade de São Paulo* (protocol number 405/11).

The animals were divided into six experimental groups, according to the solutions given during sensitization and challenge, route of sensitization used, and the doses of dust mites applied to the study groups. The six groups are detailed on table 1.

**Table 1:** Experimental groups according to the sensitization route, substances applied (saline solution or *Dermatophagoides pteronyssinus* - Der p), and doses applied

Groups	Sensitization (dose and route)	Challenge (10mcL, IN route)	n (initial)	n (final)
SalSC	Saline SC	Saline	6	6
Derp50SC	Der p 50mcg SC	Der p 50mcg	6	6
Derp500SC	Der p 500mcg SC	Der p 50mcg	6	6
SalIP	Saline IP	Saline	5	5
Derp50IP	Der p 50mcg IP	Der p 50mcg	6	5
Derp500IP	Der p 500mcg IP	Der p 50mcg	6	6

IN: intranasal; SC: subcutaneous; IP: intraperitoneal; n: number of mice.

The dust mite studied was the Der p, using lyophilized powder extract* (Alergia Clínica Laboratorial e Comércio Ltda., *São Paulo, SP, Brazil*)* diluted in 0.9% saline solution, with no preservative and stored in the refrigerator (4^°^C). The concentration of the primary allergen (Der p 1 and Der p 2) was 34mcg/mg of extract. For sensitization, the aluminum hydroxide (alum) was used as adjuvant, at the dose of 6mg per application. The study groups received three applications of Der p at the doses of 50mcg (1.7mcg of the primary allergen) or 500mcg (17mcg of the primary allergen), diluted in saline with 6mg alum, with seven-day intervals. The Control Groups received three applications of saline solution with 6mg alum, with the same interval between doses. The volume applied in each injection was 0.2mL (Table 1).

During the challenge phase, the nasal instillations contained 10mcL of the solution, with saline (Control Groups) or Der p at the dose of 50mcg, diluted in 0.9% saline solution.

**Figure 1:**
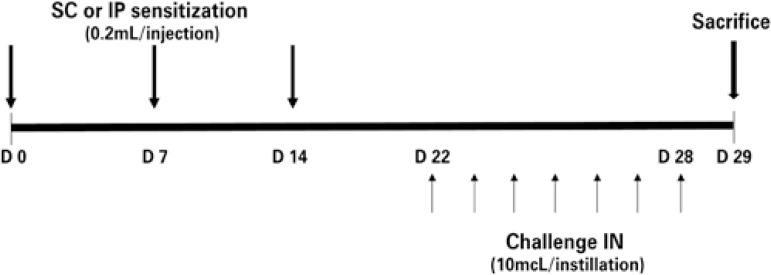
Diagram of the protocol for sensitization, challenge, and sacrifice with duration of 30 days (D0 to D29)

SC: subcutaneous; IP: intraperitoneal; IN: intranasal.

The protocol for pulmonary sensitization and inflammation induction by dust mite had the duration of 30 days, as is summarized in figure 1. The animals received the injections of the sensitizing agent on days 0.7, and 14, and were submitted to challenges with daily IN instillations between days 22 and 28; they were sacrificed for study on day 29.

The sensitization routes used were subcutaneous (SC) and IP. The SC injections were applied at the base of the tail. The animals were divided into six groups, according to the route of administration and the doses used in sensitization (Table 1). All groups received alum at the dose of 6mg.

The animals received the IN instillations of the study substance daily from day 22 to 28, between 9:00 am and 11:00 am. The SalSC and SalIP groups received 10mcL of the saline solution, while the Derp50SC, Derp500SC, Derp50IP, and Derp500IP groups were submitted to IN administration of 50mcg of Der p, diluted in saline, with a total of 10mcL.

On day 29, 24 hours after the last IN installation, the animals were anesthetized by IP injection of thiopental (70mg/kg) and then underwent tracheostomies. Still under the effect of the anesthetic, the mice were exsanguinated, via dissection of the abdominal aorta, with removal of the lungs for histological examination.

After the collection of the bronchoalveolar lavage, the lungs were removed *en bloc* to be submitted to histological analysis. The fixation of the material was done with 4% formaldehyde for 24 hours. The material was cut to posteriorly be included in paraffin and stained with hematoxylin-eosin (HE). The slides were analyzed with a common optic microscope for the determination of the mean alveolar diameter, peribronchial infiltrate, and parameters indicative of pulmonary remodeling.

The morphometric analysis was performed with the help of a reticulum with 50 lines and 100 points, with known area, coupled with the optic microscope. Hematoxylin-eosin staining was used to quantify the eosinophils in the peribronchovascular space. The density of eosinophils was determined by counting the number of eosinophils present in the inflammatory infiltrate between the bronchus and the adjacent artery, divided by the number of points, corresponding to the total area of the inflammatory infiltrate, with a magnification of 1,000X. On each slide prepared with tissue from only one animal, five airways were analyzed.

To quantify the collagen fibers present in the peribronchovascular space, the slides were stained with Picrosirius (Direct Red 80, CI 35780, Aldrich, Milwaukee, Wisc., USA). The area of the collagen fibers was measured (mm^(2)^) in five distal areas, using polarized light with a 400X magnification, with an image analyzing system (Image J, version 1.30).^(15)^ The collagen area was expressed as a relation between the area of the collagen fibers (mm^(2)^) and the perimeter of the airway (mm).

Serum from the blood collected by dissection of the abdominal aorta was used for investigation of specific anti-Der p immunoglobulins, by means of the passive cutaneous anaphylaxis (PCA) technique for IgE detection and immunoenzymatic assay (ELISA) for determination of specific anti-Der p IgG1. After blood collection, the samples were immediately centrifuged for 10 minutes (5^°^C; 3000rpm). Samples of serum were stored at -80^°^C until the day of the assays.

Passive cutaneous anaphylaxis reaction was used to detect and estimate the levels of IgE specific for Der p, as previously described.^(16,17)^ In short, the dorsum of each rat was shaved and 0.1mL of different serum solutions was injected by intradermal route. Three male Wistar rats were used for the PCA, and the serum of each animal was included in the study. After a latent period of 24 hours, the animals were challenged intravenously (IV) with 1mL of 0.5% Evans Blue solution in saline, containing 1,000mcg of the antigen (Der p). The animals were submitted to euthanasia 30 minutes after the injection of the antigen, and the diameter of the blue papules on the internal surface of the skin was measured. The PCA titers were defined as the greatest dilutions that lead to an intradermal allergic reaction greater than 5mm in diameter. The experiments were run in triplicate.

Dosing of the antibodies was done by indirect ELISA. For IgG1, the micro plaque was covered with the antigen Der p. After incubation and washing, the sera were added at a previously determined dilution. To develop the reaction, IgG1-specific detection biotinylated antibodies were added, followed by incubation and washing. Next, the developing solution containing the enzymatic conjugate of streptavidin-peroxidase, substrate, and chromogene were added.

The color reaction was read in a 490nm spectrophotometer and was proportional to the quantity of the immunoglobulins in the sample. The results were expressed as mean absorbance ± standard error of the serial dilutions of the samples of each group.

The statistical analyses were carried out by means of the SigmaStat 10.0 program (Jandel, Calif., EUA). Comparison between the groups was made with the one-way analysis of variance (ANOVA), followed by the Holm-Sidak method. A p value of less than 0.05 was considered significant.

## RESULTS

The protocol proved safe and effective in inducing chronic allergic pulmonary disease. Only one animal from Group 5 died before the 29^th^ day, which was not attributed to respiratory symptoms.

**Figure 2:**
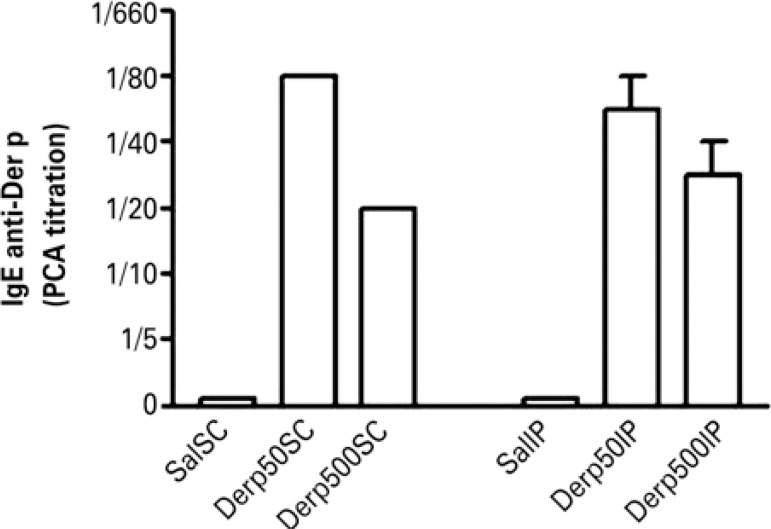
Values of mean ± standard error of the titration of specific IgE anti-*Dermatophagoides pteronyssinus* by the passive cutaneous anaphylaxis technique

Both the SC and IP sensitization routes were effective in inducing IgE specific for Der p (Figure 2). Both doses of dust mites also lead to the synthesis of specific IgE, with no difference between the two groups sensitized by IP, but with greater titration in the Derp50SC Group (1:80) relative to the Derp500SC Group (1:20), as is shown on figure 2. No animal of the Control Groups (Saline) showed positivity in the investigation of specific IgE (Figure 2).

Der p: *Dermatophagoides pteronyssinus; *PCA: passive cutaneous anaphylaxis.

**Figure 3:**
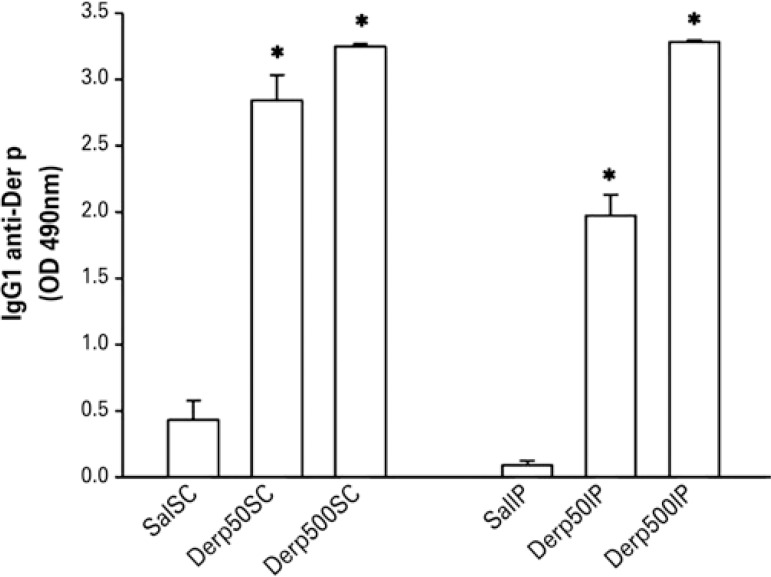
Values of mean ± standard error of the titration of specific IgG1 anti-*Dermatophagoides pteronyssinus* by the immunoenzymatic assay technique (ELISA)

The IgG1 quantified by ELISA was also detectable at high titers in the four groups submitted to the dust mite, with no difference among them (Figure 3).

* p<0.01 for the respective saline groups. Der p: *Dermatophagoides pteronyssinus.*

**Figure 4:**
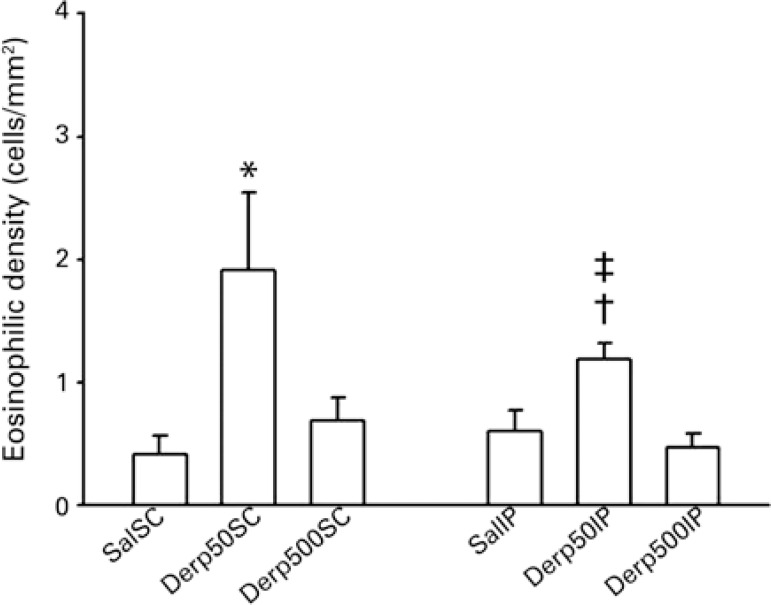
Values of mean ± standard error of the eosinophilic density in the peribronchovascular space (hematoxylin-eosin). The density was determined by the count of number of eosinophils present in the inflammatory infiltrate, between the bronchus and the adjacent artery, divided by the number of points corresponding to the total area of the inflammatory infiltrate, with a 1000x magnification

The eosinophilic inflammation in the peribronchovascular space was analyzed by cell density. Evaluating the density of eosinophils in the peribronchovascular space, there was no difference between the two sensitization routes. In the groups sensitized by the SC route, there was an increase in eosinophilia density in the Derp50SC Group relative to the SalSC Group (p=0.049). Among the groups sensitized by IP route, the Der50IP Group had a greater density of eosinophilia than did the SalIP and Derp500IP Groups (p=0.017 and 0.025, respectively). The results of the analysis of eosinophilic density are detailed in figure 4.

* p=0.049 (between groups Derp50SC and SalSC); † p=0.017 (between groups Derp50IP and SalIP); ‡ p=0.025 (between groups Derp50IP and Derp500IP).

**Figure 5:**
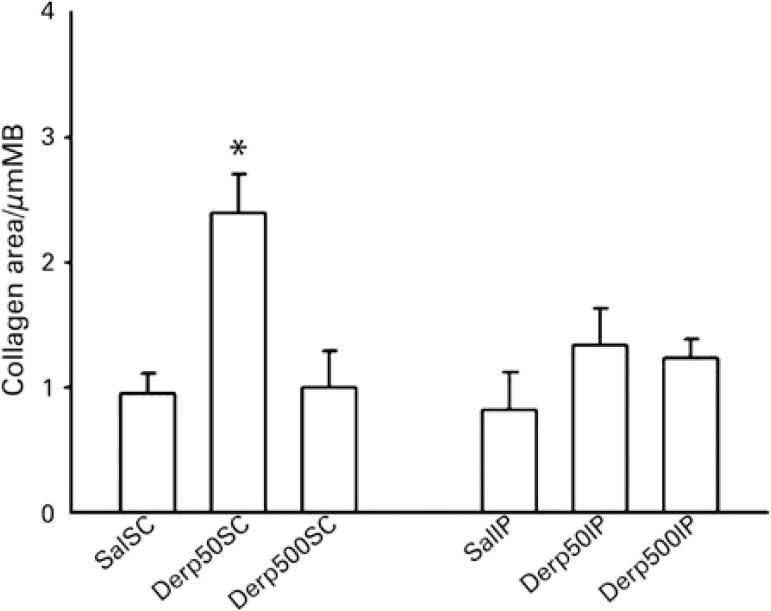
Evaluation of airway remodeling by the quantification of collagen fibers stained by Picrosirius. The collagen area (mean ± standard error) was expressed by a ratio of the area of the collagen fibers (in µm2) and the perimeter of the airway (in µm2)

Remodeling of the airways was analyzed by the area of collagen fibers relative to the perimeter of the airway. Only Group Derp50SC had an increase in collagen fibers relative to all other experimental groups**.** The intraperitoneal route and the 500mcg dose of Der p did not prove effective in inducing remodeling, showing results similar to those in the mice treated only with saline (Figure 5).

* p<0.005 (between Group Derp50SC and all the other groups). BM: basal membrane.

## DISCUSSION

Allergic asthma is a highly prevalent disease, especially in Western societies. Over the last years, there has been much progress in the understanding of the immunopathological mechanisms, and animal models have been fundamental in the study of the complex cellular and physiological interactions that occur in the human disease.^(18)^ Additionally, it is known that sensitization to house dust mites is the primary risk factor for the development of respiratory allergic diseases.^(19)^ With this, the number of studies published using dust mites in animal models has been increasing, providing a more trustworthy mimicking of the disease found in clinical practice, and a better understanding of its pathological mechanisms.

Several combinations of different routes of sensitization and challenge have already been used in murine models of asthma due to dust mites, including IP, SC, IN sensitization as well as aerosol, IN and intratracheal challenge.^(10-13,20)^ However, few studies were published with a combination of SC sensitization and IN challenge.^(10,21)^ The SC and IP routes for sensitization are comparable in models of ovalbumin,^(22)^ but as far as we know, the present study was the first to compare the SC and IP sensitization route in a model of asthma due to dust mites.

Since asthma is a disease with a TH2 profile, with an IgE-mediated mechanism, the first condition for developing an efficient model of allergic pulmonary disease is an effective sensitization, with synthesis of specific IgE at adequate levels.^(10)^ We can attain high titers of IgE, mediated by the PCA technique. We also show the formation of high titers of anaphylactic IgG1, corroborating findings of IgE. Additionally, the two sensitization routes (SC and IP) were effective, as well as the two doses studied (50 and 500mcg of Der p per injection). The groups submitted to doses of 50mcg of Der p seem to present with higher levels of specific IgE than the 500mcg groups, particularly in animals sensitized by the SC route, in which we find two different titers, which is considered significant in the PCA technique. In a prior study, our group showed that the increase in dose used in sensitization lead to an increase in IgE in BALB/c mice and decrease in C57Bl/6.^(14)^ Our results suggest the occurrence of an immunomodulation with the dose of 500mcg administered in three injections.

Another important fact is the participation of eosinophils in the pathogenesis of asthma.^(23,24)^ Eosinophils are recruited by cytokines and chemokines to the site of inflammation after degranulation of the mast cells by the allergen-IgE bond and then reach the location of the aggression, cause epithelial damage, increase the inflammatory process, and are considered the primary cause responsible for the persistent inflammatory process in the asthma patient’s airways.^(23)^ We were able to demonstrate the increase of eosinophilia in the peribronchovascular space after sensitization with 50mcg IP relative to the other groups sensitized via IP. In the groups sensitized via SC, only the group with the lower dose (Derp50SC) showed an increase in eosinophilia in the peribronchovascular space relative to its negative control (SalSC). Once again, the high dose of 500mcg per application in the SC Group may have caused a deviation of the immune response to tolerance.

Nonetheless, the most accentuated difference found in the present study was in regard to the remodeling of the airways. The Derp50SC Group showed a prominent increase in the area of collagen fibers relative to the perimeter of the airways when compared to the other groups. The 500mcg SC dose did not trigger a greater production of collagen fibers, even when compared to the Saline Group. These findings corroborate our hypothesis that the higher doses must have induced regulatory populations, diminishing the production of IgE, the allergic inflammation, and the chronification of the process. Additionally, the IP route of sensitization also did not prove efficient in inducing remodeling, both in the group that received the lower dose (50mcg) and in the group submitted to the 500mcg injections. This shows the importance of more studies such as the one presented here that compare different routes and doses for the induction and sensitization with an aeroallergen. The confirmation that the 50mcg doses were more effective than those at 500mcg not only strengthens the concept of tolerance by high doses, but also allows future studies to be done using smaller quantities of the allergen, with greater cost-efficacy.

## CONCLUSION

The model of experimental asthma with duration of 30 days, based on SC sensitization with 50mcg of Der p with aluminum hydroxide, in three applications, followed by a daily nasal challenge with Der p 50mcg in 10mcL of saline for 7 days, and euthanasia 24 hours after the last challenge, was effective in the development of specific IgE and IgG1, eosinophilia in the peribronchovascular space, and airway remodeling. The intraperitoneal route proved less effective in this study than the SC route, and higher doses of dust mites for sensitization (500mcg) were inferior to those of 50mcg, suggesting immunomodulation. More studies are needed to assess the efficacy of this model in the development of airway hyperresponsiveness, but it may be replicated in experiments for establishing new therapeutic strategies with medications or even immunotherapy.
