# Supplementary material for: Sensitization by subcutaneous route is superior to intraperitoneal route in induction of asthma by house dust mite in a murine mode
Source: Einstein (Sao Paulo). 2015 Oct-Dec;13(4):560–6. doi: 10.1590/S1679-45082015AO3389 (PMC4878631; doi:10.1590/S1679-45082015AO3389)
Supplement: Translation

# Translation: Sensibilização por via subcutânea é superior à intraperitoneal na indução de asma por ácaro em modelo murino

**Authors:** Marcelo Vivolo Aun, Beatriz Mangueira Saraiva-Romanholo, Francine Maria de Almeida, Thayse Regina Brüggemann, Jorge Kalil, Milton de Arruda Martins, Fernanda Magalhães Arantes-Costa, Pedro Giavina-Bianchi

**Affiliations:** 1 Faculdade de Medicina, Universidade de São Paulo, São Paulo SP, Brasil.

**Keywords:** Modelos animais, Asma/induzido quimicamente, Pyroglyphidae

## Abstract

**Objetivo:**

Desenvolver um novo modelo experimental de doença pulmonar alérgica crônica por ácaro, com proeminente produção de imunoglobulina E (IgE) específica, infiltrado inflamatório eosinofílico nas vias aéreas e remodelamento, comparando duas vias diferentes de sensibilização.

**Métodos:**

O protocolo teve duração de 30 dias. Camundongos BALB/c foram divididos em seis grupos submetidos à sensibilização por via subcutânea ou intraperitoneal com solução salina (controles negativos), *Dermatophagoides pteronyssinus* (Der p) 50 ou 500mcg, em três aplicações. Posteriormente, foram submetidos à provocação intranasal com Der p ou salina por 7 dias e sacrificados 24 horas após o último desafio. Avaliamos a titulação de IgE específica anti-Der p, densidade eosinofílica no espaço peribroncovascular e remodelamento das vias aéreas.

**Resultados:**

Tanto os animais sensibilizados por via subcutânea como intraperitoneal produziram IgE específica anti-Der p. Ocorreu aumento da eosinofilia peribroncovascular apenas nos animais que receberam menor dose de Der p. Porém apenas o grupo sensibilizado com Der p 50mcg subcutânea apresentou remodelamento significativo das vias aéreas.

**Conclusão:**

Neste modelo murino de asma, as duas vias de sensibilização levaram à produção de IgE específica e eosinofilia nas vias aéreas. No entanto, apenas a via subcutânea foi capaz de induzir ao remodelamento. Além disso, doses menores de Der p utilizadas foram superiores às mais elevadas, sugerindo tolerância. Mais estudos são necessários para avaliar a eficácia deste modelo no desenvolvimento da hiperresponsividade brônquica, mas ele pode ser replicado em experimentos para criação de novas estratégias terapêuticas medicamentosas ou imunoterápicas.

## INTRODUÇÃO

A asma é uma doença inflamatória das vias aéreas associada à hiperresponsividade brônquica, que leva a episódios recorrentes de tosse, dispneia, sibilância e opressão torácica.^(1,2)^ A patogênese da asma alérgica, que responde por até 70% dos casos, envolve a exposição a alérgenos, o fenômeno da sensibilização e a consequente reação de hipersensibilidade do tipo I, mediada por imunoglobulina E (IgE).^(3,4)^ No entanto, o mecanismo imunopatológico, inicialmente mediado por IgE, é muito mais complexo, incluindo diversas células, mediadores inflamatórios e citocinas, com perfil predominante de linfócitos T auxiliares ou *helper *2 (TH2).^(5)^ A complexidade da patogênese da asma humana dificulta a criação de modelos experimentais com animais que reproduzam de forma fidedigna as formas clínicas que encontramos na prática diária.^(1,6,7)^

A compreensão dos mecanismos patológicos, das vias críticas envolvidas e de alvos para novas estratégias terapêuticas seria idealmente determinada por estudos em seres humanos.^(6,7)^ Porém, por questões éticas óbvias, os estudos necessários para tais conclusões não são realizados em asmáticos humanos.^(6)^ Desse modo, a formulação de modelos animais simples, eficazes e que não demandem custo muito elevado se faz imprescindível.

Os animais mais comumente utilizados em modelos de asma têm sido os roedores, particularmente os camundongos e ratos, pela maior disponibilidade, custo-efetividade e facilidade de manuseio.^(6)^ A maioria dos modelos de asma com camundongos utiliza a ovalbumina como proteína estranha sensibilizante associada a um adjuvante, notadamente o hidróxido de alumínio.^(7)^ No entanto, modelos mais recentes vêm utilizando ovalbumina sem adjuvante para sensibilização, com resultados comparáveis aos modelos clássicos, com alume.^(8)^

A via mais utilizada para sensibilização é a intraperitoneal (IP), em geral com duas injeções, separadas por um período de 7 a 14 dias e, normalmente, ocorre nova exposição à proteína antigênica diretamente na via aérea, via inalatória (aerossol), instilação intranasal (IN) ou intratraqueal.^(7)^ Porém tem sido questionada a significância clínica de modelos com ovalbumina para mimetizar a asma humana. Dessa forma, vêm sendo utilizados modelos com ácaros, pólens, baratas ou fungos, que são conhecidos aeroalérgenos envolvidos na patogênese da asma alérgica humana.^(7,9)^ Aeroalérgenos já foram estudados para sensibilização e provocação por diferentes vias e com doses variadas.^(10-13)^ Nosso grupo já demonstrou, inclusive, que diferentes linhagens de camundongos podem apresentar respostas antagônicas em curvas dose-resposta de sensibilização ao ácaro *Dermatophagoides pteronyssinus* (Der p) em um modelo de conjuntivite alérgica.^(14)^ No entanto, uma das grandes dificuldades na montagem de modelos eficazes de asma tem sido a cronicidade do processo, com desenvolvimento de doença IgE mediada, com eosinofilia pulmonar, mas que acarrete também remodelamento das vias aéreas.

## OBJETIVO

Desenvolver um novo modelo experimental de doença pulmonar alérgica crônica por ácaro, com proeminente produção de IgE específica, infiltrado inflamatório eosinofílico nas vias aéreas e remodelamento, e comparar duas vias diferentes de sensibilização e duas doses distintas de ácaro neste processo.

## MÉTODOS

Foram utilizados camundongos machos adultos, livre de patógenos específicos (SPF, sigla do inglês *specific-pathogen-free*), da linhagem BALB/c, com idade de 6 a 8 semanas. Os animais foram provenientes do Biotério Central da Faculdade de Medicina da Universidade de São Paulo. Os camundongos foram mantidos no biotério de manutenção dos Laboratórios de Investigação Médica (LIM) 05 e 20, sob alimentação e água *ad libitum*. Todos os animais receberam cuidados de acordo com o Guia de Cuidados e Uso de Animais de Laboratório, publicado no *National Institutes of Health* (NIH; *publication* 85-23, revisado em 1985), e todos os experimentos foram realizados sob anestesia geral. O protocolo foi aprovado pelo Comitê de Ética em Pesquisa da Faculdade de Medicina da Universidade de São Paulo (protocolo 405/11).

Os animais foram divididos em seis grupos experimentais, de acordo com as soluções administradas na sensibilização e provocação, a via de sensibilização utilizada e as doses de ácaro aplicadas nos grupos de estudo. Os seis grupos estão detalhados na tabela 1.

**Tabela 1:** Grupos experimentais de acordo com a via de sensibilização, substâncias aplicadas (salina ou *Dermatophagoides pteronyssinus* - Der p) e doses aplicadas

Grupos	Sensibilização (dose e via)	Provocação (10mcL, via IN)	n (inicial)	n (final)
SalSC	Salina SC	Salina	6	6
Derp50SC	Der p 50mcg SC	Der p 50mcg	6	6
Derp500SC	Der p 500mcg SC	Der p 50mcg	6	6
SalIP	Salina IP	Salina	5	5
Derp50IP	Der p 50mcg IP	Der p 50mcg	6	5
Derp500IP	Der p 500mcg IP	Der p 50mcg	6	6

IN: intranasal; SC: subcutânea; IP: intraperitoneal; n: número de camundongos.

O ácaro estudado foi o Der p, utilizando-se extrato em pó liofilizado (Alergia Clínica Laboratorial e Comércio Ltda., São Paulo, SP, Brasil), diluído em solução salina 0,9% sem conservante e armazenado em geladeira (4°C). A concentração do alérgeno principal (Der p 1 e Der p 2) era de 34mcg/mg de extrato. Para a sensibilização, foi utilizado como adjuvante o hidróxido de alumínio (alume), na dose de 6mg por aplicação. Os grupos de estudo receberam três aplicações de Der p nas doses de 50mcg (1,7mcg de alérgeno principal) ou 500mcg (17mcg de alérgeno principal), diluídos em salina com alume 6mg, com intervalos de 7 dias. Os Grupos Controles receberam três aplicações de solução salina com alume 6mg, com o mesmo intervalo entre as doses. O volume aplicado em cada injeção foi de 0,2mL (Tabela 1).

Durante a fase de desafio ou provocação, as instilações nasais continham 10mcL da solução, com salina (Grupos Controles) ou Der p na dose de 50mcg, diluído em soro fisiológico 0,9%.

O protocolo de sensibilização pulmonar e indução da inflamação por ácaro teve a duração de 30 dias, conforme esquematizado na figura 1. Os animais receberam as injeções do agente sensibilizante nos dias 0,7 e 14, sendo submetidos às provocações com instilações IN diárias entre os dias 22 e 28 e sacrificados para estudo no dia 29.

**Figura 1:** Esquema do protocolo de sensibilização, provocação e sacrifício com duração de 30 dias (D0 a D29)

SC: subcutânea; IP: intraperitoneal; IN: intranasal.

As vias de sensibilização utilizadas foram a subcutânea (SC) e IP. As injeções SC foram aplicadas na base da cauda. Os animais foram divididos em seis grupos, segundo a via e as doses utilizadas na sensibilização (Tabela 1). Todos os grupos receberam alume na dose de 6mg.

Os animais receberam as instilações IN da substância de estudo do dia 22 a 28, diariamente, entre 9h e 11h. Os grupos SalSC e SalIP receberam 10mcL de solução salina, enquanto os grupos Derp50SC, Derp500SC, Derp50IP e Derp500IP foram submetidos à administração IN de 50mcg de Der p, diluído em salina, totalizando 10mcL.

No dia 29, 24 horas após a última instilação IN, os animais foram anestesiados por injeção IP de tiopental (70mg/kg) e, em seguida, traqueostomizados. Ainda sob efeito anestésico, os camundongos foram exsanguinados, via dissecção da aorta abdominal, com remoção dos pulmões para análise histológica.

Após a coleta do lavado broncoalveolar, os pulmões foram removidos em bloco para serem submetidos à análise histológica. A fixação do material foi feita com formaldeído 4%, por 24 horas. O material foi cortado para posteriormente ser incluído em parafina e corado com hematoxilina e eosina (HE). As lâminas foram analisadas em microscópio óptico comum para a determinação do diâmetro alveolar médio, infiltrado peribrônquico e parâmetros indicativos de remodelamento pulmonar.

A análise morfométrica foi realizada com o auxílio de um retículo de 50 retas e 100 pontos, com área conhecida, acoplado à ocular do microscópio ótico. A coloração com HE foi utilizada para quantificação de eosinófilos no espaço peribroncovascular. A densidade de eosinófilos foi determinada pela contagem do número de eosinófilos presentes no infiltrado inflamatório entre o brônquio e a artéria adjacente, dividido pelo número de pontos, correspondendo à área total do infiltrado inflamatório, num aumento de 1.000X. Em cada lâmina preparada com tecido de apenas um animal, cinco vias aéreas foram analisadas.

Para quantificar as fibras de colágeno presentes no espaço peribroncovascular, as lâminas foram coradas com *Picrosirius* (*Direct Red* 80, CI 35780, Aldrich, Milwaukee, Wisc., EUA). Foi medida a área de fibras colágenas (mm^(2)^) em cinco vias aéreas distais, utilizando-se luz polarizada no aumento de 400X, com um sistema analisador de imagem (*Image* J, versão 1.30).^(15)^ A área do colágeno foi expressa em uma relação entre a área das fibras colágenas (mm^(2)^) e o perímetro da via aérea (mm).

O soro do sangue coletado pela dissecção da aorta abdominal foi utilizado para a pesquisa de imunoglobulinas específicas anti-Der p, por meio da técnica da anafilaxia cutânea passiva (PCA - *passive cutaneous anaphylaxis*) para detecção de IgE e de ensaio imunoenzimático (ELISA) para determinação de IgG1 específicas anti-Der p. Após a coleta do sangue, as amostras foram imediatamente centrifugadas por 10 minutos (5^°^C; 3000rpm). Amostras do soro foram armazenadas a -80^°^C até o dia da realização dos ensaios.

A reação da PCA foi usada para detectar e estimar os níveis de IgE específicas para Der p, como descrito previamente.^(16,17)^ Brevemente, o dorso de ratos foi depilado e 0,1mL de diferentes diluições do soro foi injetado por via intradérmica. Três ratos machos da linhagem Wistar foram usados para realizar a PCA, e o soro de cada animal foi incluído no estudo. Após um período latente de 24 horas, os animais foram desafiados por via intravenosa (IV) com 1mL de solução de azul de Evans a 0,5% em salina, contendo 1.000mcg do antígeno (Der p). Os animais foram submetidos à eutanásia 30 minutos após a injeção do antígeno e o diâmetro das pápulas azuis na superfície interna da pele foi medido. Os títulos do PCA foram definidos como as maiores diluições que levaram a uma reação alérgica intradérmica maior do que 5mm de diâmetro. Os experimentos foram realizados em triplicata.

A dosagem de anticorpos foi realizada por ELISA indireto. Para determinação de IgG1, a microplaca foi recoberta com antígeno Der p. Após incubação e lavagem, os soros foram adicionados em uma diluição previamente determinada. Para revelar a reação, foram adicionados anticorpos de detecção biotinilados específicos para IgG1, seguido de incubação e lavagem. A seguir, a solução reveladora contendo conjugado enzimático de estreptavidina-peroxidase, substrato e cromógeno foi adicionada.

A reação de cor foi lida em espectrofotômetro a 490nm e foi proporcional à quantidade das imunoglobulinas na amostra. Os resultados foram expressos como a média das absorbâncias ± erro padrão das diluições seriadas das amostras de cada grupo.

As análises estatísticas foram realizadas por meio do programa *SigmaStat* 10.0 (Jandel, Calif., EUA). A comparação entre os grupos foi feita com análise de variância simples (*One Way* ANOVA), seguida pelo método Holm-Sidak. Foi considerado significante p menor que 0,05.

## RESULTADOS

O protocolo mostrou-se seguro e eficaz na indução da doença pulmonar alérgica crônica. Apenas um animal do Grupo 5 evoluiu a óbito antes do 29º dia, o que não foi imputado a sintomas respiratórios.

Tanto as vias de sensibilização SC como IP foram eficazes na indução da produção de IgE específica para Der p (Figura 2). As duas doses de ácaro utilizadas também levaram à síntese de IgE específica, sem diferença entre os dois grupos sensibilizados por via IP, mas com maior titulação no Grupo Derp50SC (1:80) em relação ao Derp500SC (1:20), como mostrado na figura 2. Nenhum animal dos Grupos Controle (Salina) apresentou positividade na pesquisa de IgE específica (Figura 2).

**Figura 2:** Valores de média ± erro padrão da titulação da IgE específica anti-*Dermatophagoides pteronyssinus* pela técnica da *passive cutaneous anaphylaxis*

Der p: *Dermatophagoides pteronyssinus;* PCA: passive cutaneous anaphylaxis.

A IgG1 quantificada por ELISA também foi detectável em altos títulos nos quatro grupos submetidos ao ácaro, sem diferença entre eles (Figura 3).

**Figura 3:** Valores de média ± erro padrão da titulação da IgG1 específica anti-* Dermatophagoides pteronyssinus* pela técnica da ensaio imunoenzimático (ELISA)

*p<0,01 para respectivos grupos salina. Der p: *Espermatozoide pteronyssinus.*

A inflamação eosinofílica no espaço peribroncovascular foi analisada pela densidade de células. Avaliando a densidade de eosinófilos no espaço peribroncovascular, não houve diferença entre as duas vias de sensibilização. Nos grupos sensibilizados por via SC, houve aumento da densidade eosinofílica no Grupo Derp50SC em relação ao Grupo SalSC (p=0,049). Entre os grupos sensibilizados por via IP, o Grupo Der50IP teve maior densidade eosinofílica do que os Grupos SalIP e Derp500IP (p=0,017 e 0,025, respectivamente). Os resultados da análise da densidade eosinofílica estão detalhados na figura 4.

**Figura 4:** Valores de média ± erro padrão da densidade eosinofílica no espaço peribroncovascular (hematoxilina e eosina). A densidade foi determinada pela contagem do número de eosinófilos presentes no infiltrado inflamatório, entre o brônquio e a artéria adjacente, dividido pelo número de pontos correspondendo à área total do infiltrado inflamatório, num aumento de 1000x

* p=0,049 (entre grupos Derp50SC e SalSC); † p=0,017 (entre grupos Derp50IP e SalIP); ‡ p=0,025 (entre grupos Derp50IP e Derp500IP).

O remodelamento das vias aéreas foi analisado pela área de fibras colágenas em relação ao perímetro da via aérea. Apenas o Grupo Derp50SC teve aumento das fibras colágenas em relação a todos os outros grupos experimentais**.** A via intraperitoneal e a dose de 500mcg de Der p não se mostraram eficazes na indução de remodelamento, tendo resultados semelhantes aos dos camundongos tratados apenas com salina (Figura 5).

**Figura 5:** Avaliação do remodelamento das vias aéreas pela quantificação de fibras colágenas coradas por Picrosirius. A área do colágeno (média ± erro padrão) foi expressa em uma relação entre a área das fibras colágenas (em µm2) e o perímetro da via aérea (em µm2)

* p<0,005 (entre Grupo Derp50SC e todos os demais grupos). MB: membrana basal.

## DISCUSSÃO

A asma alérgica é uma doença de alta prevalência, especialmente nas sociedades ocidentais. Nos últimos anos, houve muito progresso na compreensão dos mecanismos imunopatológicos, e os modelos animais têm sido fundamentais no estudo das complexas interações celulares e fisiológicas que ocorrem na doença humana.^(18)^ Além disso, sabe-se que a sensibilização aos ácaros da poeira é o principal fator de risco para desenvolvimento de doenças alérgicas respiratórias.^(19)^ Com isso, vem aumentando o número de trabalhos publicados utilizando ácaros em modelos animais, de modo a mimetizar de forma mais fidedigna a doença encontrada na prática clínica e compreender melhor seus mecanismos patológicos.

Várias combinações de diferentes vias de sensibilização e provocação já foram utilizadas em modelos murinos de asma por ácaros, entre elas sensibilização por via IP, SC, IN e provocação aerossol, IN e intratraqueal.^(10-13,20)^ Porém poucos foram os estudos publicados com a combinação de sensibilização SC e provocação IN.^(10,21)^ As vias SC e IP para sensibilização são comparáveis em modelos de ovalbumina,^(22)^ mas, até onde sabemos, o presente estudo foi o primeiro a comparar as vias de sensibilização SC e IP em um modelo de asma por ácaro.

Como a asma é uma doença de perfil TH2, com mecanismo IgE-mediado, a primeira condição para desenvolver um modelo eficiente de doença pulmonar alérgica é uma sensibilização eficaz, com síntese de IgE específica em níveis adequados.^(10)^ Conseguimos altos títulos de IgE, medidos pela técnica de PCA. Mostramos também formação de altos títulos de IgG1 anafilática, corroborando achados da IgE. Além disso, as duas vias de sensibilização (SC e IP) foram eficazes, bem como as duas doses estudadas (50 e 500mcg de Der p por injeção). Os grupos submetidos a doses de 50mcg de Der p parecem ter apresentado níveis mais elevados de IgE específico do que os grupos 500mcg, particularmente nos animais sensibilizados por via SC, nos quais encontramos diferença de duas titulações, o que é considerado significante na técnica de PCA. Em estudo prévio, nosso grupo demonstrou que o aumento da dose utilizada na sensibilização levava a aumento da IgE em camundongos BALB/c e diminuição em C57Bl/6.^(14)^ Nossos resultados sugerem a ocorrência de uma imunomodulação com a dose de 500mcg administrada em três injeções.

Outro dado importante é a participação do eosinófilo na patogênese da asma.^(23,24)^ O eosinófilo é recrutado por citocinas e quimiocinas para o sítio da inflamação após a desgranulação do mastócito ativado pela ligação alérgeno-IgE e, depois de chegar ao local da agressão, causa dano epitelial, aumento do processo inflamatório e é considerado o principal responsável pelo processo inflamatório persistente na via aérea do asmático.^(23)^ Conseguimos demonstrar o aumento da eosinofilia no espaço peribroncovascular após a sensibilização com 50mcg IP em relação aos outros grupos sensibilizados via IP. Nos grupos sensibilizados por via SC, apenas o de dose menor (Derp50SC) apresentou aumento da eosinofilia peribroncovascular em relação ao seu controle negativo (SalSC). Mais uma vez, a dose elevada de 500mcg por aplicação no Grupo SC pode ter acarretado desvio da resposta imune para tolerância.

No entanto, a diferença mais marcante encontrada no presente estudo se deu em relação ao remodelamento das vias aéreas. O Grupo Derp50SC apresentou um aumento proeminente da área de fibras colágenas em relação ao perímetro das vias aéreas quando comparado aos outros grupos. A dose de 500mcg por via SC não desencadeou maior produção de fibras colágenas nem mesmo em comparação ao Grupo Salina. Esses achados corroboram nossa hipótese de que as doses mais altas devem ter induzido populações regulatórias, diminuindo a produção de IgE, à inflamação alérgica e à cronificação do processo. Além disso, a via de sensibilização IP também não se mostrou eficiente na indução de remodelamento, tanto no grupo que recebeu a dose menor (50mcg) como no que foi submetido às injeções de 500mcg. Isso mostra a importância de mais estudos como o aqui apresentado, que comparem vias e doses diferentes para induzir à sensibilização com aeroalérgeno. A comprovação de que as doses de 50mcg foram mais eficazes que as de 500mcg não só fortalece o conceito de tolerância por altas doses, mas permite que estudos futuros sejam realizados utilizando menores quantidades de alérgeno, com maior custo-eficácia.

## CONCLUSÃO

O modelo de asma experimental com duração de 30 dias, baseado na sensibilização SC com 50mcg de Der p com hidróxido de alumínio, em três aplicações, seguidas por provocação nasal diária com Der p 50mcg em 10mcL de salina por 7 dias, e sacrifício após 24 horas do último desafio foi eficaz no desenvolvimento de IgE e IgG1 específicas, eosinofilia no espaço peribroncovascular e remodelamento das vias aéreas. A via intraperitoneal mostrou-se menos eficaz neste estudo do que a SC e doses mais elevadas de ácaro para sensibilização (500mcg) foram inferiores às de 50mcg, sugerindo imunomodulação. Mais estudos são necessários para avaliar a eficácia deste modelo no desenvolvimento da hiperresponsividade das vias aéreas, mas ele pode ser replicado em experimentos para criação de novas estratégias terapêuticas com medicamentos ou mesmo imunoterapia.
